# Care Pathways and Determinants in Essure® Contraceptive Implant Use and Removal: A Qualitative Study

**DOI:** 10.1111/hex.70534

**Published:** 2026-04-24

**Authors:** Jéronine Tengue, Adeline Darlington‐Bernard, Titouan Michon, Nathalie Bernoud‐Hubac, Benoit Ter‐Ovanessian, Ana‐Maria Trunfio‐Sfarghiu, Claude Dussart, Romain Lan, Florence Carrouel

**Affiliations:** ^1^ Laboratory “Health, Systemic, Process” (P2S), UR4129 University Claude Bernard Lyon 1, University of Lyon Lyon France; ^2^ INSA Lyon, CNRS, LaMCoS, UMR5259 Villeurbanne France; ^3^ INSA Lyon, CNRS, MatéIS, UMR5510 Villeurbanne France; ^4^ Hospices Civils de Lyon Lyon France; ^5^ Laboratory Anthropologie Bio‐Culturelle, Droit, Ethique et Santé (ADES), Centre National de la Recherche Scientifique (CNRS), Etablissement Français du Sang (EFS) Aix Marseille University Marseille France

**Keywords:** adverse effect, hysterectomy, pain, qualitative methodology, quality of life, sterilisation, women health

## Abstract

**Background:**

Introduced in 2002, the Essure® implant was promoted as a minimally invasive permanent contraceptive. After widespread use, increasing reports of adverse effects led to its global withdrawal in 2018. This study explores the experiences of women in France who chose Essure®, focusing on their motivations and the factors leading to its removal.

**Methods:**

A qualitative descriptive study was conducted in 2024, involving 17 semi‐structured interviews to document participants' clinical pathways, implantation experiences and decision‐making around removal. Interviews were recorded, transcribed verbatim and analysed thematically. The study followed the COREQ checklist and received ethical approval (reference 2024‐04‐04‐005).

**Results:**

All participants reported symptom onset following implantation, including chronic joint, muscle and abdominal pain. Complications such as tubal damage, device migration and breakage during removal were also described. Beyond physical symptoms, women experienced psychological distress and social consequences. Many reported inadequate pre‐procedural counselling, particularly regarding the device's composition and potential risks. Participants often encountered insufficient support from healthcare providers when reporting symptoms or requesting removal.

**Conclusion:**

The study highlights the broad impact of Essure®, revealing not only physical complications but also emotional and social burdens. Findings underscore the need for comprehensive informed consent, empathetic communication and responsive care in the management of contraceptive device complications.

**Patient or Public Contribution:**

Women with lived experience of Essure® implantation and removal were central to this study. They were involved in shaping the research question and informing the design through preliminary interviews, which contributed to refining the interview guide. Participants provided rich, first‐hand narratives through semi‐structured interviews, allowing for an in‐depth understanding of clinical pathways, complications and the psychosocial impact of the device. Recruitment was conducted in collaboration with the RESIST patient association, whose support was instrumental in identifying eligible participants. The study thus reflects patient‐led priorities and experiential knowledge, contributing to the interpretation of findings and the development of patient‐centred recommendations. Their involvement did not end at the preliminary stage. The experiential input from these women was also later mobilised to reorient the interpretation of emerging findings during the thematic analysis, ensuring that analytical categories remained anchored in lived experience.

## 1 Background

Contraception is a public health measure aimed at reducing unwanted pregnancies and remedying abortions, which can have a negative impact on women's social and professional opportunities [1]. According to the United Nations, in 2019, 76% of women of childbearing age (15–49 years), equivalent to 23.7% of all women, or approximately 219 million, used contraception, with sterilisation being the most common method worldwide [2].

Between 2010 and 2013, female sterilisation rates in France showed a modest increase due to the combined effects of post‐2001 legal reforms [3, 4], declining trust in hormonal contraceptives following the 2012 ‘pill scare’ [3], improved access to minimally invasive sterilisation techniques [4] and the national reimbursement of Essure® implants, which significantly reduced financial barriers to hysteroscopic sterilisation [5]. However, a decline followed between 2013 and 2022, with procedures dropping from 45,138 to 20,325 [6]. A 2015 report from the French National Agency for Medicines and Health Product Safety (ANSM) revealed that among 38,533 women using sterilisation as contraception, 23,711 had chosen the Essure® method [7, 8]. France ranked second after the United States in absolute numbers of Essure® implantations and was the first country where this method surpassed surgical tubal ligation [9].

Presented as the future of permanent contraception, Essure® devices were implanted into nearly 200,000 women in France between 2002 and 2017 [10]. These medical devices offered advantages such as the absence of incisions and the possibility of insertion via the vaginal route without general anaesthesia and in ambulatory care [11]. Essure® was a permanent contraceptive device composed primarily of a nickel–titanium alloy (nitinol), stainless steel, polyethylene terephthalate (PET) fibres, tin solder and platinum–iridium markers. It was inserted hysteroscopically into the fallopian tubes, where it triggered a localised inflammatory response that led to fibrosis and tubal occlusion over the course of 3 months [12]. However, they have been associated with adverse effects, which have since caused lasting harm to thousands of patients. Indeed, their daily lives are now affected by adverse effects: chronic fatigue, depression, endocrine disorders, digestive issues, balance problems, as well as speech and memory impairments [13]. Additionally, the hypothesis of nickel allergies has been reported [14, 15, 16, 17], along with muscle and joint pain [18, 19, 20], device migrations [21, 22, 23], and contraceptive failures [9]. A French study based on data from hospitals and the ANSM in 2018, comparing more than 71,000 women sterilised with the Essure® implant to 34,000 who underwent tubal ligation, revealed an increased risk of gynaecological complications 1 and 3 years after Essure® implantation [24]. Another study conducted among patients at the Amiens University Hospital in 2018 revealed a 15.4% prevalence of adverse effects following the implantation of Essure® devices [25]. Further research analysing fallopian tube or uterine horn tissues post‐implantation highlighted significant corrosion of the device at the tin solder and the presence of tin‐rich metallic microparticles in the surrounding tissues [26, 27]. This deterioration was found to cause granulomatous inflammation in the tissues of numerous participants. Moreover, certain systemic cognitive disorders could be linked to the dispersion and accumulation of tin particles in other parts of the body, potentially transforming into organotin, a toxic form of tin [28].

In 2015, class‐action lawsuits were filed in the United States against the Essure® system, prompting the Food and Drug Administration (FDA) to launch a safety evaluation of the device [29]. Following this, the ANSM began enhanced monitoring of the implant in France. By 2017, despite a sharp increase in reported adverse effects (rising from 42 in 2012 to 242 in 2015), the Temporary Specialized Scientific Committee (CSST) concluded that ‘the literature, monitoring data, and results of the epidemiological study did not call into question the favourable benefit‐risk balance of the medical device’ [30]. However, following a collective action led by patients highlighting the risks associated with the device, its commercialisation was suspended in 2018 [31].

Essure® implants are no longer marketed globally [27, 32], but many women with these devices continue to be monitored in hospitals and health centres as they seek scientific explanations for their concerns. After implant placements were officially stopped, the number of explantation surgeries increased significantly among affected women. In France, 30,000 women out of nearly 200,000 women who received the implant had it removed between 2017 and 2020 [33]. However, although studies indicate that implant removal alleviates most symptoms and significantly improves patients' quality of life, some symptoms appear to persist, including chronic pain, fatigue, endocrine and digestive disorders, balance disturbances, and cognitive impairments such as memory or speech difficulties [13, 18, 19, 20].

Despite the data available on adverse effects and symptom alleviation following explantation, a lack of detailed insights into the personal experiences and decision‐making processes of women regarding Essure® implantation and removal remains.

This study aimed to analyse and model the care pathways of women in France who chose Essure® as a contraceptive method, including those who later opted for its removal, while identifying the determinants influencing their decisions regarding implantation and removal.

## 2 Methods

### 2.1 Justification of the Method

According to the literature, most studies conducted to understand the health scandal surrounding Essure® implants were epidemiological in nature [34]. This approach did not allow for an exploration of the personal experiences of the patients affected. In this work, a qualitative study based on interviews was chosen. This methodological approach is appropriate to ‘understand the world from the subjects' perspective’ and to explore their lived experiences in a sensitive health context [35]. Additionally, qualitative research interviews allow for the collection of subjective information about the participants, and more specifically, their experiences [36]. Semi‐structured interviews, in particular, provide a framework to analyse and compare participants' responses effectively [37]. Including women who have had these implants both inserted and removed is crucial to capture the full spectrum of experiences and decision‐making processes, shedding light on factors influencing both adoption and explantation.

Although the study was primarily descriptive, its analytical framework was grounded in a phenomenological approach, which aims to explore how individuals perceive, experience and make sense of their lived reality within a particular social and temporal context. This orientation seeks to capture the essence of subjective experiences rather than to generate generalisable findings. It was therefore particularly appropriate for understanding the perceptions, emotions and decision‐making processes of women who underwent Essure® implantation and removal [38, 39, 40].

### 2.2 Study Design

This is a qualitative study with a descriptive design focused on the care pathways of participants with the Essure® device. It was conducted from 1 October to 13 December 2024. The qualitative analysis report was in compliance with the Consolidated Criteria for Reporting Qualitative Research (COREQ) recommended by the EQUATOR Network (Enhancing the QUAlity and Transparency of Health Research) (Supplementary File 1) [41].

### 2.3 Study Sample

Inclusion criteria were women: (i) aged 18 years or older, (ii) who had Essure® implants between 2002 and 2018, (iii) who had removed Essure® implants, (iv) who provided free and informed consent to participate in the study and (v) who authorised the recording and processing of their voice.

The non‐inclusion criteria were women: (i) who opposed the use of their data for research purposes, (ii) who had difficulties expressing themselves in French, (iii) who were protected or incarcerated and (iv) who had severe disabilities.

### 2.4 Sampling

Recruitment was carried out by sending an email through the French RESIST association (a support, information and mutual aid network for tubal sterilisation) [42]. Women interested in participating in the study contacted the laboratory via email. Each participant received an information notice electronically, which included details about the nature and purpose of the study, the data collected, the physical or legal bodies which would receive the data, and their rights to access, rectify or oppose the processing of their data. Participants were also given a 7‐day reflection period to decide whether to take part in the study. They had no information regarding the researcher.

Thus, the study used a purposive sampling strategy [43], targeting women who had experienced both the implantation and removal of the Essure® device. Since participation was voluntary following the RESIST association's call, the recruitment also included elements of convenience sampling [44], consistent with qualitative research aiming for depth and richness of information rather than statistical representativeness.

### 2.5 Sample Size

In qualitative research, the number of participants is not predetermined; participant recruitment is stopped upon reaching data saturation, as the number of individuals interviewed is not a determining factor for the validity of the results. Data saturation, marked by redundancy, serves as the primary indicator. Thus, an initial sample size is estimated, but the final sample size is determined by data saturation [45].

In fact, several qualitative research experts have shared their perspectives on this matter, emphasising that there is no straightforward answer to the question of ‘how many’ participants are needed [46]. Recent methodological studies emphasise that qualitative samples should be sufficiently rich to generate new, textured insights while remaining small enough to allow in‐depth analysis [47, 48]. Morse suggests that the more usable the data collected from each participant, the fewer participants are required [49]. Similarly, Lincoln and Guba propose that sample size determination should follow the principle of redundancy, meaning that sampling can cease when no new information is obtained from additional units [50].

In our study, interviews were stopped after reaching data saturation, which occurred after the 17th interview.

### 2.6 Development of the Interview Guide

The development of the guide could not rely on pre‐existing or validated models from previous studies, as a thorough literature review revealed none. Consequently, the research team designed the guide based on a consensus‐building process. Consensus methods are systematic approaches to synthesising information and reconciling conflicting viewpoints to establish the level of agreement within a selected group [51]. In this case, the Delphi method was employed to achieve a final, unified and convergent opinion within the group [52, 53]. The group of experts consisted of healthcare professionals and public health specialists. Each member independently generated a list of questions they deemed relevant to explore the care pathways of women who had chosen Essure® as a contraceptive method. These individual proposals were then consolidated into a single set of questions, which underwent an initial rating round to evaluate their suitability for inclusion in the interview guide. The group of experts rated the questions on a scale from 1 (completely disagree) to 9 (completely agree), with 5 indicating indecision. Ratings were analysed according to the criteria outlined in Supplementary File 2. Questions deemed appropriate were included in the interview guide, while those rated as inappropriate were excluded. For questions with uncertain ratings or missing values, the group engaged in a second round of discussion and re‐evaluation. If consensus could not be achieved during the second round, the question was ultimately excluded from the guide.

This guide was piloted in two individual telephone interviews with women who had chosen Essure® as their contraceptive method but who did not take part in our study. Their feedback, based on lived experience, allowed us to refine the wording and focus of several questions, ensuring that the guide reflected women's perspectives and experiential priorities. These interviews allowed us to collect suggestions on the questions and adapt the guide [54, 55].

The interview guide was organised in three sections (Supplementary File 3). The first introductory section aimed to assess the participants' current health status and their overall condition at the time of the interview. This section also gathered contextual information on the temporality of their care and follow‐up, including the timing of Essure® implant removal and the duration for which the implants were worn. The second section addressed the key elements and determinants of care pathways leading to the decision to have Essure® implants inserted. The objective was to identify the critical factors influencing the care pathways of all the women who opted for Essure® implants. The third section explored the determinants of care pathways specific to the women who decided to have their Essure® implants removed, aiming to uncover the factors influencing their decision and subsequent care. The interview guide concluded with questions aimed at collecting the participants' socio‐demographic data (age, education level, and profession before and after the removal). Occupation was classified according to the INSEE (French National Institute of Statistics and Economic Studies) ‘Professions and Socioprofessional Categories’ nomenclature [56].

### 2.7 Data Collection

Data collection was carried out through interviews held in France between 1 October and 13 December 2024. The interviews were conducted by a female researcher (co‐author) by videoconference with the Cisco WebEx application or phone, depending on the preference of the participant. The interviewer is a Master of Science in Public Health student, trained in qualitative studies. No relationship was established with the participants prior to the start of the study. Each interview began with an explanation of the procedure, data protection and anonymisation measures, and the purpose of the interview. The interviewer guided the discussions according to the interview guide, using prompts or requests for rephrasing to probe topics further. This interview guide was used to ensure consistency while allowing participants to express their experiences in depth [57]. Women with lived experience also contributed to validating the relevance of questions and confirming that their perspectives were accurately reflected in the data collection process. All interviews, conducted face‐to‐face between the interviewer and the participant, were recorded on WebEx, transcribed verbatim (using the Noota software) and then deleted. Transcript accuracy was reviewed by another trained qualitative co‐author researcher. No notes were taken during and/or after the interview.

### 2.8 Data Analysis

Data analysis was conducted using a hypothetico‐deductive approach and coding methods. The five‐step methodology framework for thematic analysis, as proposed by Braun and Clarke, was applied [58].

1. Familiarisation with the data: This step involved reading and re‐reading the transcripts to fully immerse in and understand the content.
2. Generating initial codes: Raw data (verbatim transcripts) were transformed into initial meaningful codes. These were then organised into themes aligned with the research questions and hypotheses. In a first step of decontextualisation, relevant excerpts were isolated to create semantically independent units for thematic grouping. This was followed by recontextualisation to restore meaning across grouped codes and themes.
3. Searching for themes: The data were reorganised through a hierarchical structure of concepts to generate broader themes. These themes were enriched and interconnected via constant comparison, ultimately forming a structured framework based on the initial research questions. During this stage, the coding framework was reflexively reviewed against the experiential elements recurrently emphasised by participants. This ensured that themes were not only derived from a deductive structure suggested by professionals but genuinely grounded in women's lived trajectories and meaning‐making processes.
4. Reviewing themes: Themes were reviewed and refined collaboratively.
5. Defining and naming themes: Finally, finer coding was used to illustrate the themes with specific examples. At least one verbatim is selected for each theme.

### 2.9 Data Validation

To enhance the credibility and reliability of findings, several validation strategies were applied. First, transcripts were systematically verified by a second researcher to ensure accuracy. Data analysis was conducted by two coders who compared and discussed emerging themes until consensus was reached, thereby reinforcing dependability. In addition, data saturation was used as a criterion to determine sample adequacy. Triangulation helped validate the consistency and relevance of the themes identified. Participant quotes are presented to illustrate themes, allowing readers to evaluate the connection between raw data and interpretation. Participants did not review the final themes, but their experiential input was used iteratively to guide and refine data interpretation throughout the analysis.

### 2.10 Reflexivity Statement

The research team acknowledges the potential influence of positionality on the collection and interpretation of data. Interviews were conducted by a trained female researcher, not directly involved in the medical management of Essure®, which facilitated openness while limiting hierarchical bias. Nevertheless, as public health researchers and clinicians, our prior knowledge of Essure® controversies may have shaped the analytical lens. To mitigate this, reflexive discussions were held throughout the study to critically examine assumptions and ensure that women's narratives were prioritised as the main interpretive framework.

### 2.11 Ethical Approval

The study received ethical approval from the Research Ethics Committee of the University (reference 2024‐04‐04‐005) on 20 June 2024. All participants received both written and verbal information about the study and gave their informed consent to participate.

## 3 Results

### 3.1 Description of the Study Population

Table 1 presents the socio‐demographic characteristics of the 17 study participants. The average age at the time of Essure® implantation was 40.35 ± 3.75 years, 50.29 ± 5.42 years at the time of removal and 52.76 ± 4.89 years at the time of the interview. The mean duration of implant use was 9.94 ± 4.35 years.

**Table 1:** Characteristics of the participants.

Variable	
Age	Mean ± SD years
At the time of implantation	40.35 ± 3.75
At the time of removal	50.29 ± 5.42
At the time of the interview	52.76 ± 4.89

Education level (*N* = 17)	*n* (%)
< High school diploma (BAC)	1 (5.88)
[High school diploma (BAC) – BAC + 2]	4 (23.53)
[Bachelor's (BAC + 3) – Master's (BAC + 5)]	11 (64.71)
> Master's (BAC + 5)	1 (5.88)

Occupation (*N* = 17)	*n* (%)
Executives and higher intellectual professions	8 (47.06)
Intermediate professions and mid‐level executives	6 (35.29)
Employees	3 (17.65)

Among the participants, 11 (64.71%) had an education level ranging from a 3‐year degree (BAC + 3) to a Master's (BAC + 5), 8 (47.06%) belonged to the category of executives and higher intellectual professions, and 6 (35.29%) belonged to the category of intermediate professions and mid‐level executives.

### 3.2 Thematic Analysis

Interviews (average duration 41.38 ± 6.65 min) were subjected to inductive coding and thematic analysis, which revealed three primary steps in participants' care trajectories: (i) Pathway and determinants leading to Essure® implantation, (ii) Pathway and determinants leading to implant removal and (iii) Experiences and consequences post‐removal. These stages encompassed a total of nine themes (Table 2).

**Table 2:** Summary table of thematic analysis.

Step	Theme	Sub‐theme
Step 1: Pathway and determinants for the implantation of Essure® implants	Theme 1: Decision‐making process for permanent contraception	Factors influencing the initial decision to choose permanent contraception
		Factors influencing the choice of Essure®
	Theme 2: Information and awareness deficits about Essure® implants	Lack of information on placement risks
		Lack of information about the composition
		Lack of information about potential complications
Step 2: Pathway and determinants leading to implant removal	Theme 3: Device‐related complications during insertion	Tubal injury
		Dual implantation
	Theme 4: Long‐term impact on health and well‐being	Physical health issues
		Psychological consequences
		Social and relational impact
		Device‐related complications
	Theme 5: Delayed awareness of risks and emotional reactions.	Awareness post‐insertion through personal means
		Negative emotional reactions
		Critique of pharmaceutical influence and regulatory failure
	Theme 6: Interaction with healthcare professionals	Perceived lack of support from healthcare professionals during the implant removal process
		Difficulties in accessing appropriate healthcare for diagnosing health issues
		Logistical challenges in obtaining implant removal
	Theme 7: Navigating information and care pathways for implant removal	Role of Patient Associations and Media Networks
		Medical guidance and the importance of adhering to protocol
		Gaps in medical support and the burden of self‐advocacy
Step 3: Pathways and determinants after implant removal	Theme 8: Difficulties in accessing post‐operative care	Lack of comfort measures during hospital stay
		Barriers to coordinated home‐based care
	Theme 9: Symptoms resolution and long‐term concerns	Improvement after implant removal
		Persistent or emerging symptoms
		Fear and uncertainty about long‐term health

### 3.3 Pathway and Determinants for the Placement of Essure® Implants

#### 3.3.1 Decision‐Making Process for Permanent Contraception

- Factors influencing the initial decision to choose permanent contraception

Participants cited several reasons for choosing permanent contraception, including:

− Ending childbearing

Most women explained they chose permanent contraception because they no longer wished to have children:

My partner and I decided that we didn′t want any more children.

P10

− Medical considerations

For some, health risks or contraindications to hormonal contraception prompted the choice:

My gynecologist told me: “Because of your history of phlebitis, you can no longer take hormones, so the pill is not an option.” She then suggested Essure®.

P7

− Unsuitable alternatives

Others turned to Essure® after experiencing problems with other contraceptive methods:

The copper IUD didn′t work for me; it caused excessively heavy bleeding. As a midwife, I had heard about the Essure® implant at conferences, and I thought, “When I turn 42, this might be the right solution for me.”′

P3

- Factors influencing the choice of Essure®

Women's choice of the Essure® method was shaped by healthcare professionals, including gynaecologists, surgeons and midwives, who presented the device and its procedure in a reassuring and non‐invasive manner.

− Trust in medical discourse

Many interviewees reported high trust in the reassurances offered by healthcare providers:

I trusted the medical professionals.

P13

The gynecologist described it to me as a safe, revolutionary procedure. In theory, no anesthesia, no outpatient procedure, no surgery, and you could resume your normal life the very next day.

P8

They told me: “There's no risk with the anesthesia. Unlike tubal ligation, you go home the same day, it doesn′t hurt, it just feels a bit like period cramps.”’

P4

− Perceived Safety of the device

The composition of the implant was also described in reassuring terms, contributing to the perception of safety:

The surgeon told me it's just a titanium device, it's very safe and there are no risks’

P6

### 3.4 Information and Awareness Deficits About Essure® Implants

Several women reported not receiving full or clear information from healthcare professionals prior to the procedure.

- Lack of information on placement risks

Some interviewees were unaware of potential technical complications during insertion, such as misplacement or tissue damage:

I wasn't told that during the placement, it could puncture something, be improperly positioned, or end up too far in or too far out. I wasn't even informed that there could be an issue with the placement itself.

P13

- Lack of information about the composition

Participants expressed the lack of information regarding the device's materials:

They never provided the full composition. When I asked the gynecologist what Essure® was made of, he responded, “Why does that matter to you?”

P12

- Lack of information about potential complications

Women also highlighted the absence of information about possible adverse outcomes, such as migration:

There was zero information. He never mentioned that it could migrate or move out of the fallopian tubes, for example.

P10

### 3.5 Pathway and Determinants Leading to Implant Removal

#### 3.5.1 Device‐Related Complications During Insertion

Some participants reported complications during the placement, including a damaged fallopian tube or the insertion of an additional implant:

They damaged one of my fallopian tubes during the placement.

P11

After the procedure, the gynecologist came to see me in my room and said: “Listen, there was a bit of an issue. I thought I hadn't managed to catch the fallopian tube, so I inserted an extra implant. You now have two on the left side and one on the right.”

P5

#### 3.5.2 Long‐Term Impact on Health and Well‐Being

Interviewees mentioned significant physical, psychological and social effects:

- Physical health issues

Several participants described persistent physical symptoms, including chronic pain, gynaecological symptoms, fatigue and loss of professional capacity:

I had joint pain, a number of pains that were severe enough to wake me up at night and prevent me from doing certain things.

P9

After they placed the implants, I bled a lot; I kept bleeding for 4 months. I spent over 2 years having heavy, hemorrhagic periods

P16

I had a lot of vaginal yeast infections.

P12

After the implant placement, working was no longer possible.

P15

I became disabled because of the implant. […] I ended up in a wheelchair. For 8 years, I lost my job.

P14

- Psychological consequences

Alongside physical symptoms, several women reported they experienced psychological distress, including episodes of depression and mental exhaustion:

I asked for antidepressants because I was feeling depressed. Mentally, it was difficult. The symptoms were extremely intense; I even felt at one point that I was going to die.

P10

- Social and relational impact

The burden of these health complications often extended to participants' personal lives, with some talking about significant relational tensions or breakdowns:

The implant ruined my relationship with my daughters. It also led to my breakup with my partner at the time. It wasn't without consequences; it was truly difficult and heartbreaking.

P2

### 3.6 Device‐Related Complications

Certain participants experienced complications directly related to the device itself, such as implant migration and misplacement:

One of my implants migrated it ended up in the left iliac crest area, near my intestine.

P9

The implant I lost, I think it must have been poorly positioned.

P3

### 3.7 Delayed Awareness of Risks and Emotional Reactions

- Awareness post‐insertion through personal means

Women acknowledged that awareness of the risks associated with the Essure® implant only emerged after its insertion, primarily through personal research, patient associations and media coverage:

Now I have the information. I know that if it's poorly placed or if there's an issue, it can cause real physical pain because I researched the subject and learned about the dangers related to the composition of the implants.

P16

- Negative emotional reactions

Obtaining information on their own led to emotional reactions marked by anger, betrayal and mistrust:

I would have liked to have been told a little bit about what the side effects might be or what conditions might or might not develop after insertion. When the problems started to become known (…) I was very angry.

P1

- Critique of pharmaceutical influence and regulatory failure

Interviewees criticised pharmaceutical influence and questioned institutional oversight:

I believe there was strong political backing that enabled this device to be approved for the market. We're talking about “lobbies”, pharmaceutical companies, with a lot of money‐making profit from women's health…

P11

### 3.8 Interaction With Healthcare Professionals

- Perceived lack of support from healthcare professionals during the implant removal process

Most participants reported a lack of support, empathy and attentiveness from healthcare professionals when they sought to have the implants removed, making the process even more difficult:

When I talked about removal, the gynecologist who followed me was in complete denial. She told me that, in her opinion, there was no point in removing them but added, “If it helps you psychologically, go ahead, but it won't change anything, this is not what caused all your health issues.”

P14

I saw a neurologist who basically treated me like I was crazy. Even my gynecologist didn't believe that removing the implants was necessary. My general practitioner was also against it, he wasn't interested in it, he told me it was just a minor health issue. The medical community rejected me so much…

P3

- Difficulties in accessing appropriate healthcare for diagnosing health issues

Several women highlighted experiencing ‘medical wandering’, characterised by repeated tests and consultations without receiving a clear diagnosis. This often worsened their health and delayed appropriate treatment:

The steps that led me to have it removed were the diagnostic uncertainty surrounding a state of generalized fatigue in a body that was no longer able to respond to the call [i.e., a body that no longer had the energy or functional capacity to perform daily activities or cope with basic physical demands].

P6

- Logistical challenges in obtaining implant removal

The lack of support and awareness among healthcare professionals regarding Essure® complications created significant obstacles. Some participants had to travel far from home to access specialised care:

When I realized that in my region, I wouldn′t find any leads, support, help, or even an appointment with someone who could guide me, it was chaos […]. So, I discovered that I would have to travel to other cities where two specialized facilities had doctors who were actually addressing this problem.

P11

### 3.9 Navigating Information and Care Pathways for Implant Removal

Faced with limited or inconsistent information from healthcare providers, participants described diverse strategies to access knowledge and support for Essure® implant removal.

- Role of Patient Associations and Media Networks

Patient associations and social media groups played a critical role in informing and empowering participants. These communities not only provided detailed practical advice but also connected women with others who had undergone explantation, helping them find competent specialists and feel less isolated:

I contacted association X and was able to get all the information about explantation techniques and the side effects. They connected me with other patients who had already undergone explantation, and these women shared their stories and gave me the names of the doctors who had operated on them. That's how I found my way forward.

P5

- Medical guidance and the importance of adhering to protocol

Some interviewees recounted positive experiences with healthcare professionals who were knowledgeable about the national removal protocol and took the time to explain the surgical process. These clinicians provided reassurance and helped patients feel more secure in their decision to pursue explantation:

The surgeon explained that great care had to be taken to avoid breaking the implants and that he was very familiar with the national protocol. He drew many diagrams to show how he would proceed, where he would cut.

P11

- Gaps in medical support and the burden of self‐advocacy

However, other participants stated that they received no guidance from healthcare providers and had to rely solely on their own research:

The healthcare professionals didn′t provide me with any information it was actually me who took the initiative, thanks to my own research.

P16

In cases where the removal protocol was not followed, complications occurred. One woman had to undergo a second surgery due to implant fragmentation:

After undergoing an X‐ray, I discovered that two fragments were still present on either side of the uterus; the implants had been broken in two during the first surgery.

P8

### 3.10 Pathways and Determinants After Implant Removal

#### 3.10.1 Difficulties in Accessing Post‐Operative Care

- Lack of comfort measures during hospital stay

Participants reported insufficient pre‐operative guidance regarding post‐surgical discomfort, noting the absence of practical comfort measures such as heated blankets to alleviate pain, especially gas‐related pain after laparoscopy:

There are so many comfort measures for the first few days after the procedure that are not included in the general list of what we should bring during our stay to ease the pain, for example, to deal with gas from the laparoscopy or having heated blankets.

P12

- Barriers to coordinated home‐based care

Upon discharge, interviewees encountered logistical and organisational difficulties accessing follow‐up care, particularly for tasks like post‐operative injections. Many felt unprepared for the complexity of arranging home visits or obtaining nursing support, leaving them to manage their recovery alone:

Then there's the issue of care once we're discharged; in the end, we don't anticipate how complicated it will be to get appointments with nurses for injections, so we end up having to do them ourselves. Many of us find ourselves in this situation.

P12

### 3.11 Symptoms Resolution and Long‐Term Concerns

Experiences after implant removal varied.

- Improvement after implant removal

Some participants reported significant health improvements:

Today I am much, much better than I was a year ago. I weigh 10 kilos more than I did a year ago. I have resumed a normal life and can once again give my children the attention they need.

P9

I've never felt this good. I haven't regained all my previous energy, but I'd say I'm at least at 60 out of 100. For me, it feels almost like a miracle.

P6

- Persistent or emerging symptoms

Others experienced persistent symptoms, particularly pain and cognitive disorders:

I still have pain. I can feel some lingering muscle tension in my abdomen. As for joint pain, it's a bit hard to say because I still have it.

P5

Today, I still have memory and concentration problems.

P15

In some cases, new conditions emerged after removal, such as Biermer's disease (pernicious anaemia), although a direct link with the device could not be established.

We don't know if Biermer's disease developed because of the implant. I had no prior psychiatric history, and no one in my family has Biermer's disease.

P17

- Fear and uncertainty about long‐term health

Many women expressed ongoing concern about their long‐term health and the unknown risks of the device:

Even though I no longer have any symptoms today, since I underwent a total hysterectomy with ovary removal, I still have poison in my blood. What will happen? Could I develop new conditions? An autoimmune disease? Cancer? I have no answers, but these are my fears. And since no one is monitoring me to find out, I'm navigating in complete uncertainty.

P1

## 4 Discussion

The aim of this study was to explore the experiences of women in metropolitan France who received the Essure® implant between 2002 and 2017 and later opted for explantation. The analytical lens adopted in this study was intentionally co‐constructed, drawing initially from a professional consensus framework, but was then actively reshaped by the experiential narratives shared by participants. This iterative process aligns with participatory qualitative standards, fostering an interpretive framework grounded in patient knowledge rather than exclusively biomedical categories. While existing literature often emphasises clinical outcomes or procedural safety, our work adopts a holistic, patient‐centred approach. It captures not only the biomedical harms, but also the emotional, social and existential toll taken by the Essure® experience. The emergence of activism and advocacy among some patients illustrates the transition from suffering to empowerment, a phenomenon rarely discussed in prior studies.

The results of this study revealed a complex landscape of lived experiences characterised by adverse physical symptoms, limited access to information and gaps in medical follow‐up, especially concerning the removal process. These experiences highlight significant discrepancies between patient expectations, clinical communication and actual care received, particularly during the post‐implantation and explantation phases.

Consistent with previous reports, the majority of participants in this study experienced symptoms following implantation. These included chronic joint and muscular pain, fatigue, and gynaecological complications, such as heavy menstrual bleeding, recurrent infections and persistent pelvic discomfort. Our results align with those of Zou et al. (2023) [59], who reported diffuse, pelvic and abdominal pain, haemorrhagic complications, and autoimmune‐like symptoms, including fibromyalgia, and with findings from Leleu A. et al. (2021) [60] and Zegura A. et al. (2022) [61], who observed pelvic and musculoskeletal pain as common concerns. Similarly, Insubri S. et al. (2024) [27] identified joint and muscular pain as the most frequently reported complaints among patients.

Beyond the physical sequelae, our study underscores the profound psychological and social impact of adverse experiences with the Essure® implant. Participants described emotional distress, including depression and anxiety, as well as disruptions to family life, employment and social integration. These psychosocial consequences remain under‐represented in the existing literature but are crucial for a holistic understanding of the impact of the device. While Zou C. et al. (2023) [59] did not specifically address psychosocial dimensions, they did report cases of disability and hospitalisations related to Essure®, which underscores the potential severity of its consequences.

Furthermore, our findings suggest that the decision to choose Essure® was not solely patient‐driven but was often strongly influenced by healthcare professionals. Several participants emphasised the pivotal role of gynaecologists, surgeons and midwives in presenting Essure® as a superior, modern and non‐invasive option, often without a balanced comparison to alternative methods. The high level of trust participants placed in the medical profession appears to have shaped their decisions, particularly when clinicians minimised potential risks or emphasised perceived benefits. This reflects a form of informational bias, where the framing of Essure® as a safe and routine procedure may have contributed to limited critical engagement with the decision‐making process. Such dynamics highlight the importance of ensuring genuinely informed consent, based on comprehensive and unbiased counselling. However, it is equally important to recognise that the responsibility of healthcare professionals may have been significantly shaped by systemic gaps in the dissemination of safety information. With regard to the implantation procedure, the first specific warning notices about associated risks were only shared with surgeons in 2016 [62], despite Essure® being introduced in 2002 and alerts to the ANSM as early as 2003. At that time, reporting to health authorities remained limited, with only 26 alerts per year received by the ANSM before January 2013 [63]. Regarding the guidelines for surgical implant removal, although a warning was issued in 2018, the official notice targeting both patients and healthcare providers was not distributed until 2023 [64]. These delays in the circulation of crucial safety information likely constrained practitioners' ability to fully inform and protect patients. This context of fragmented knowledge underscores the need for more transparent, timely and centralised medical communication systems.

Beyond informational and systemic shortcomings, our findings also reveal a relational dimension: several participants perceived the attitude of healthcare professionals as dismissive, condescending or emotionally distant when reporting symptoms or requesting implant removal. This perceived lack of listening and empathy reflects broader discussions on gender bias and medical paternalism in reproductive health, where women's complaints are often underestimated or attributed to psychological rather than physiological causes. Similar findings have recently been documented in qualitative studies addressing women's experiences of chronic pelvic pain, endometriosis, vulvovaginal disorders and maternity care, notably in the United Kingdom and other European settings [65, 66, 67, 68]. These results underline the need for a more patient‐centred and gender‐sensitive approach to reproductive healthcare communication and practice.

In terms of complications, our findings include implant migration, structural breakage during removal, and cases of tubal or uterine damage during insertion. These are in line with reports from Zou C. et al. (2023) [59], who documented severe complications such as uterine perforations, intra‐abdominal bleeding and even cancer‐related mortality. Conversely, studies such as Câmara S. et al. (2017) [69] found no serious acute complications. Their long‐term follow‐up revealed high patient satisfaction, with only a few isolated cases of nickel allergy and unilateral expulsion. This variation underscores the need to contextualise our findings within the broader landscape of patient responses. The heterogeneity in experiences may reflect differences in patient selection, insertion technique, follow‐up care and individual predispositions. Regarding post‐explantation outcomes, the majority of participants in our study reported partial or full resolution of symptoms. However, in some cases, certain symptoms, particularly chronic pain, persisted, complicating recovery and quality of life. These findings are consistent with those of Chene G. et al. (2019) [70] and Insubri S. et al. (2024) [27], who reported variable improvements in post‐operative well‐being, with some patients experiencing rapid relief and others noting continued distress.

Although the Essure® device is no longer marketed or used in France, the experiences of the women affected offer valuable lessons for improving patient safety and the quality of care in the field of reproductive health and medical device regulation.

Firstly, healthcare professionals need to ensure transparent and comprehensive communication about the composition of any contraceptive device, as well as associated risks and alternatives. Inadequate informed consent in the case of Essure® has been associated with patient distress and feelings of betrayal [71, 72, 73, 74, 75].

Secondly, when contraceptive devices are in use, standardised post‐implantation monitoring protocols could be considered to detect complications early. In the case of Essure®, complications such as device migration and systemic symptoms were often diagnosed late, sometimes resulting in irreversible harm [76].

Thirdly, the professionals involved in the insertion or removal of contraceptive devices need to receive appropriate and ongoing training that extends beyond technical knowledge and includes patient‐centred communication strategies. A lack of preparation among some clinicians in managing Essure®‐related complications contributed to breakdowns in care [69].

Finally, the healthcare system could consider establishing formal processes to include patient feedback in both clinical practice and health policy. Testimonies from Essure® users reveal a gap between biomedical assessments and lived experiences, particularly regarding the psychological and social consequences [77, 78, 79].

This study presents important insights into the experiences of women who underwent Essure® implantation and subsequent explantation. One of the strengths of this study lies in the integration of women's perspectives not only as data but as a structuring component of the research process, from the refinement of the interview guide to the interpretive phase of analysis. However, several limitations need to be acknowledged. First, the use of a qualitative methodology, while appropriate for exploring subjective experiences and meanings, inherently limits the generalisability of findings. Participant inclusion was guided by the principle of data saturation rather than a predefined sample size, which is consistent with qualitative research standards but restricts extrapolation to the broader population of Essure® users. Second, the recruitment process may have introduced selection bias. Participants were primarily recruited via a single advocacy network and email outreach, which may have attracted individuals who had negative experiences or were more motivated to share their stories. This could result in an over‐representation of adverse outcomes and under‐representation of neutral or positive experiences with the device. Including a more diverse range of recruitment sources, such as general medical records or family planning clinics, might have allowed for a broader spectrum of perspectives. Third, interviewer bias is a recognised challenge in qualitative studies. Although interviewers were trained in qualitative interviewing techniques, their presence and intervention to maintain conversational flow may have subtly influenced participant responses, particularly through tone, phrasing or empathetic cues. While every effort was made to minimise these effects, they remain an inherent limitation of the design. Fourth, the retrospective nature of the study may have affected the accuracy of participants' recollections, particularly for those whose procedures took place over a decade ago. Memory distortions, emotional framing or evolving perspectives over time could influence the way experiences were narrated. Fifth, the study focuses exclusively on women who experienced complications or chose explantation, omitting those who may have had positive or uneventful outcomes with the Essure® implant. This selective lens, while valuable for understanding the device's risks and shortcomings, does not provide a complete picture of its overall safety and acceptability. Finally, due to French legal constraints [80], information on participants' ethnicity or race was not collected. As most participants were likely of European descent, this limits the generalisability of our results to more ethnically diverse groups.

Future research could benefit from a mixed‐methods approach, integrating both qualitative and quantitative data from a representative cohort, including those who retained the device without reported issues. Such a design would allow for a more comprehensive assessment of both benefits and risks and may inform improved guidelines for patient selection, counselling and follow‐up.

## 5 Conclusion

This study highlighted the complexity and individuality of the trajectories experienced by women who received the Essure® implant in France. While not all users encountered difficulties, the accounts of certain women reveal significant physical, psychological and emotional repercussions both during and after the use of the device. These findings underscore the need to better acknowledge and address the experiences of those who encountered adverse effects. It is essential to expand research to include these psychosocial dimensions and to develop care practices that are more attentive, inclusive and tailored to individual needs. Strengthening healthcare professional training and improving patient information are crucial to ensuring more ethical and responsive reproductive healthcare.

## Author Contributions

**Jéronine Tengue:** investigation, data curation, writing – original draft. **Adeline Darlington‐Bernard:** data curation, writing – review and editing. **Titouan Michon:** writing – review and editing. **Nathalie Bernoud‐Hubac:** writing – review and editing. **Benoit Ter‐Ovanessian:** writing – review and editing. **Ana‐Maria Trunfio‐Sfarghiu:** writing – review and editing. **Claude Dussart:** writing – review and editing. **Romain Lan:** methodology, investigation, data curation, writing – original draft. **Florence Carrouel:** conceptualisation, methodology, data curation, writing – original draft. All authors have read and agreed to the published version of the manuscript.

## Funding

The authors received no specific funding for this work.

## Ethics Statement

The study received ethical approval from the Research Ethics Committee of the University of Lyon (reference 2024‐04‐04‐005) on 20 June 2024.

## Consent

All participants gave their informed consent to participate. All participants received both written and verbal information about the study.

## Conflicts of Interest

The authors declare no conflicts of interest.

## Supporting information

ESSURE SUPPORTING FILES 131025.

## Data Availability

All data are available upon request to the corresponding author.
