# Supplementary material for: Care Pathways and Determinants in Essure® Contraceptive Implant Use and Removal: A Qualitative Study
Source: Health Expect. 2026 Apr 24;29(2):e70534. doi: 10.1111/hex.70534 (PMC13108407; doi:10.1111/hex.70534)
Supplement: Supplementary file 1 — ESSURE SUPPORTING FILES 131025. [file HEX-29-e70534-s001.docx]

**SUPPLEMENTARY FILES**

**Supplementary file 1:** Consolidated criteria for reporting qualitative studies (COREQ): 32-item checklist

| **No. Item** | **Guide questions/description** | **Reported on Page #** |
| --- | --- | --- |
| **Domain 1: Research team and reﬂexivity** |  |  |
| *Personal Characteristics* |  |  |
| 1. Interviewer/facilitator | Which author/s conducted the interview or focus group? | 8 |
| 2. Credentials | What were the researcher’s credentials? E.g. PhD, MD | 8 |
| 3. Occupation | What was their occupation at the time of the study? | 8 |
| 4. Gender | Was the researcher male or female? | 8 |
| 5. Experience and training | What experience or training did the researcher have? | 8 |
| *Relationship with participants* |  |  |
| 6. Relationship established | Was a relationship established prior to study commencement? | 8 |
| 7. Participant knowledge of the interviewer | What did the participants know about the researcher? e.g. personal goals, reasons for doing the research | 6 |
| 8. Interviewer characteristics | What characteristics were reported about the interviewer/facilitator? e.g. Bias, assumptions, reasons and interests in the research topic | 8 |

| **Domain 2: study design** |  |  |
| --- | --- | --- |
| *Theoretical framework* |  |  |
| 9. Methodological orientation and Theory | What methodological orientation was stated to underpin the study? e.g. grounded theory, discourse analysis, ethnography, phenomenology, content analysis | 9 |
| *Participant selection* |  |  |
| 10. Sampling | How were participants selected? e.g. purposive, convenience, consecutive, snowball | 6 |
| 11. Method of approach | How were participants approached? e.g. face-to-face, telephone, mail, email | 6 |
| 12. Sample size | How many participants were in the study? | 6-7 |
| 13. Non-participation | How many people refused to participate or dropped out? Reasons? | NA |
| *Setting* |  |  |
| 14. Setting of data collection | Where was the data collected? e.g. home, clinic, workplace | 8 |
| 15. Presence of non-participants | Was anyone else present besides the participants and researchers? | 9 |
| 16. Description of sample | What are the important characteristics of the sample? e.g. demographic data, date | 10-11 |
| *Data collection* |  |  |
| 17. Interview guide | Were questions, prompts, guides provided by the authors? Was it pilot tested? | 7-8 |
| 18. Repeat interviews | Were repeat inter views carried out? If yes, how many? | 8 |
| 19. Audio/visual recording | Did the research use audio or visual recording to collect the data? | 8 |
| 20. Field notes | Were ﬁeld notes made during and/or after the interview or focus group? | 9 |
| 21. Duration | What was the duration of the interviews or focus group? | 11 |
| 22. Data saturation | Was data saturation discussed? | 6-7 |
| 23. Transcripts returned | Were transcripts returned to participants for comment and/or correction? | 9 |
| **Domain 3: analysis and ﬁndings** |  |  |
| *Data analysis* |  |  |
| 24. Number of data coders | How many data coders coded the data? | 10 |
| 25. Description of the coding tree | Did authors provide a description of the coding tree? | 11-12 |
| 26. Derivation of themes | Were themes identiﬁed in advance or derived from the data? | 9 |
| 27. Software | What software, if applicable, was used to manage the data? | NA |
| 28. Participant checking | Did participants provide feedback on the ﬁndings? | 9 |
| *Reporting* |  |  |
| 29. Quotations presented | Were participant quotations presented to illustrate the themes/ﬁndings? Was each quotation identiﬁed? e.g. participant number | 12-20 |
| 30. Data and ﬁndings consistent | Was there consistency between the data presented and the ﬁndings? | 12-20 |
| 31. Clarity of major themes | Were major themes clearly presented in the ﬁndings? | 12-20 |
| 32. Clarity of minor themes | Is there a description of diverse cases or discussion of minor themes? | 12-20 |

**Supplementary file 2:** Conditions for obtaining an agreement between experts according to median value and distribution of the quotations.

| **Proposal estimated** | Degree of agreement | Median | Minimum; Maximum |
| --- | --- | --- | --- |
| **Appropriate** |  |  |  |
|  | Strong | ≥ 7 | 7; 9 |
|  | Relative | ≥ 7 | 5; 9 |
| **Inappropriate** |  |  |  |
|  | Strong | ≤ 3 | 1; 3 |
|  | Relative | ≤ 3.5 | 1; 5 |
| **Uncertain** |  |  |  |
|  | Indecision | 4 ≤ median ≤ 6.5 | 1; 9 |
|  | No consensus | All other situations | All other situations |

**Supplementary file 3:** Semi-structured interview guide.

Socio-demographic data collected:

- age,
- education level
- profession before the removal
- profession after the removal

| **Topics** | **Questions** | **Informations to collect** |
| --- | --- | --- |
| Introduction | How are you feeling at the moment? | - Presence of symptoms  - Type of symptoms/pain  - Physical and mental health |
|  | When did you have your implants removed? How long did you wear them? | - Notion and temporality of care and follow-up |
| Care pathways and determinants for Essure^®^ implant insertion | Could you describe the different stages and elements that led you to have Essure^®^ implants fitted? | - Decision-making steps  - Decision-making elements  - Identification of decision paths |
|  | - Who were the people (professionals and non-professionals) involved in your decision to have implants Essure^®^ ?  - Could you describe the main role these people played in your decision? | - Type of people involved  - Role of people involved |
|  | - What information were you given when you decided to have implants placed Essure^®^ ?  Were you informed of the treatment you would receive, and of possible complications?  - Do you feel you were sufficiently informed? If not, how did you feel about this lack of information?  - In your opinion, what information should you have been given? Why or why not? | - Information provided by healthcare professionals to support decision-making  - Perception of information level and quality  - Expectations regarding the type of information provided |
| Care pathways and determinants in Essure^®^ implant removal | Could you describe the different steps and elements that led you to have Essure^®^ implants removed? | - Reasons for withdrawal  - Decision-making elements  - Identification of decision paths |
|  | - Who were the people (professionals and non-professionals) who played a role in your decision to have the implants removed Essure^®^ ?  - Could you describe the role of these people in your choice? | - Type of people involved  - Role of people involved |
|  | - What information were you given about implant removal Essure^®^ ?  - Were you informed about the treatment you would receive and any possible complications?  - Do you feel that you were sufficiently informed? If not, how did you feel about this lack of information?  - What information do you think you should have been given? Why or why not? | - Information provided by healthcare professionals to support decision-making  - Perception of information level and quality  - Expectations regarding the type of information provided |
